# Extended dual antiplatelet therapy with ticagrelor 60 mg in patients with prior myocardial infarction: The design of ALETHEIA, a multi‐country observational study

**DOI:** 10.1002/clc.23702

**Published:** 2021-08-08

**Authors:** Eva Lesén, Christopher Hewitt, Evangelos Giannitsis, Jonatan Hedberg, Tomas Jernberg, Dimitra Lambrelli, Aldo P. Maggioni, Jason C. Simeone, Albert Ariza‐Solé, Robert F. Storey, Jurrien ten Berg, Marc Bonaca, ALETHEIA study team

**Affiliations:** ^1^ BioPharmaceuticals Medical, AstraZeneca Gothenburg Sweden; ^2^ BioPharmaceuticals Medical, AstraZeneca Cambridge UK; ^3^ University of Heidelberg Heidelberg Germany; ^4^ Department of Clinical Sciences Danderyd Hospital, Karolinska Institute Stockholm Sweden; ^5^ Real‐World Evidence, Evidera Inc. London UK; ^6^ ANMCO Research Center Florence Italy; ^7^ Real‐World Evidence, Evidera Inc. Waltham Massachusetts USA; ^8^ Hospital Universitari de Bellvitge Barcelona Spain; ^9^ Department of Infection, Immunity and Cardiovascular Disease, University of Sheffield Sheffield UK; ^10^ St Antonius Center for Platelet Function Studies, St Antonius Hospital Nieuwegein Netherlands; ^11^ University of Colorado Anschutz School of Medicine, CPC Clinical Research/CPC Community Health Aurora Colorado USA

**Keywords:** dual antiplatelet therapy, myocardial infarction, observational study, ticagrelor

## Abstract

**Introduction:**

Clinical guidelines recommend extended treatment with dual antiplatelet therapy (DAPT) with ticagrelor 60 mg (twice daily) beyond 12 months in high‐risk patients with a history of myocardial infarction (MI) who have previously tolerated DAPT and are not at heightened bleeding risk. However, evidence on patterns of use and associated clinical outcomes in routine clinical practice is limited.

**Methods:**

ALETHEIA is an observational, multi‐country study, designed to describe characteristics, treatment persistence, and bleeding and cardiovascular (CV) outcomes in post‐MI patients who initiate ticagrelor 60 mg in routine clinical practice (NCT04568083). The study will include electronic health data in the United States (US; Medicare, commercial claims) and Europe (Sweden, Italy, United Kingdom, Germany). Characteristics will be described among patients with and without ticagrelor 60 mg ≥1 year post‐MI. Assuming an a priori threshold of 5000 person‐years on‐treatment is met, to ensure sufficient precision, clinical outcomes (bleeding and CV events) among patients treated with ticagrelor 60 mg will be assessed. Risk factors for clinical outcomes and treatment discontinuation will be assessed in patients with ticagrelor 60 mg and meta‐analysis used to combine estimates across databases. Cohort selection will initiate from the ticagrelor 60 mg US and European approval dates and end February 2020. An estimated total of 7250 patients prescribed ticagrelor 60 mg are expected to be included.

**Discussion:**

An increased understanding of patterns of ticagrelor 60 mg use and associated clinical outcomes among high‐risk patients with a prior MI is needed. The a priori specified stepwise approach adapted in this observational study is expected to generate useful evidence for clinical decision‐making and treatment optimization.

## 1 INTRODUCTION

Ischemic heart disease is the leading cause of death worldwide, with myocardial infarction (MI) being the most common form.^1^ Despite optimal secondary prevention therapies, the risk of experiencing another major adverse cardiovascular (CV) event (MACE) in patients who are event‐free 1 year after MI is about 20% in the subsequent 3 years.^2^

Dual antiplatelet therapy (DAPT) with acetylsalicylic acid (ASA) and a P2Y_12_ inhibitor (e.g., ticagrelor, clopidogrel, prasugrel) reduces the risk of CV events.^3^, ^4^, ^5^ Several studies have demonstrated a reduced risk of CV events (e.g., stroke, MI, vascular death, stent thrombosis) in patients treated with DAPT following an acute coronary syndrome (ACS), but with an increased risk of major bleeding when compared to ASA or a P2Y_12_ inhibitor alone.^4^, ^6^ In patients with prior MI or coronary stenting, extended DAPT (i.e., for more than 12 months) is also associated with a significant reduction in the risk of CV events, but a higher risk of major bleeding, compared to ASA alone.^4^, ^5^, ^7^

The P2Y_12_ inhibitor ticagrelor, in combination with low‐dose ASA, is indicated for the prevention of CV events in patients with an ACS or a history of MI. The PEGASUS‐TIMI 54 trial demonstrated a lower risk of MACE with extended DAPT with ticagrelor 60 mg (twice daily) and ASA compared to ASA alone (HR 0.84; 95% CI 0.74, 0.95) in patients with a history of MI.^6^ However, an increased risk of major bleeding versus ASA alone was also observed (HR 2.32; 95% CI 1.68, 3.21). Similarly, the DAPT study demonstrated that extending DAPT with either clopidogrel or prasugrel and ASA for up to 30 months following insertion of a drug‐eluting stent for ACS or stable CAD reduced the risk of MACE (HR 0.71; 95% CI 0.59–0.85) and stent thrombosis (HR 0.29; 95% CI 0.17–0.48), compared to 12 months of DAPT, albeit with an increased risk of moderate or severe bleeding (2.5% vs. 1.6%, *p* = .001).^5^ Clinical guidelines recommend extending DAPT, with ticagrelor 60 mg preferred over clopidogrel or prasugrel, beyond 12 months in high‐risk patients with a history of MI without previous bleeding complications or conditions associated with high‐risk of major bleeding.^8^, ^9^, ^10^

It is well known that patients treated in routine clinical practice may differ from those included in clinical trials, although there are limited observational data on patients initiating ticagrelor 60 mg post‐MI.^11^ Data from routine clinical practice are increasingly recognized as a source of valuable information about patterns of medication use as well as safety and effectiveness profiles of medications used in large, heterogenous patient populations, as a complement to randomized clinical trials.^12^ With growing attention on observational studies, the use of high‐quality electronic health data (EHD) from routine clinical practice and the application of scientifically robust methodology is crucial to ensure that the evidence generated is of sufficient quality to inform decision‐making, as also highlighted by the recent initiatives from regulatory agencies in the United States (US) and Europe.^13^, ^14^

This paper presents the rationale and design of ALETHEIA, an observational study with the primary objective to describe patient characteristics, treatment persistence, and event rates of bleeding requiring hospitalization in a large multi‐country cohort of patients treated with ticagrelor 60 mg after an MI. Additional objectives include to describe event rates for a CV composite outcome (hospitalization for MI or stroke, and all‐cause mortality), and to describe treatment persistence and event rates in patient subgroups. In addition, baseline risk factors associated with bleeding and CV events, as well as with treatment discontinuation, will be explored. To contextualize the characteristics of patients initiating ticagrelor 60 mg, characteristics will also be described for patients treated with a P2Y_12_ inhibitor other than ticagrelor (clopidogrel, prasugrel, or ticlopidine), as well as patients not treated with any P2Y_12_ inhibitor at a similar point in time from their MI as observed among patients initiating ticagrelor 60 mg.

## 2 METHODS

### 2.1 Study design

ALETHEIA is an observational, multi‐country study using retrospective EHD extracted from multiple sources in the US and in several European countries (Germany, Italy, Sweden, and the United Kingdom [UK]). The full list of objectives is presented in Table 1. The study is registered at www.clinicaltrials.gov (NCT04568083).

**TABLE 1:** Study objectives

Objectives	
Primary	To describe the demographic, clinical, and treatment characteristics of patients initiating ticagrelor 60 mg treatment, at the time of their qualifying MI and at the time of treatment initiation (index date). To describe the persistence and, where feasible, adherence, to treatment with ticagrelor 60 mg, including treatment discontinuation and treatment switch. To describe the cumulative incidence and event rates (incidence rate and all‐event rate) of bleeding requiring hospitalization in patients treated with ticagrelor 60 mg using an on‐treatment approach.^a^
Secondary	To describe the cumulative incidence and event rates (incidence and all‐event rates) of the composite of MI, stroke, and all‐cause mortality in patients treated with ticagrelor 60 mg using an on‐treatment approach.^a^ To describe treatment persistence, event rates of bleeding requiring hospitalization, event rates of the composite of MI, stroke, and all‐cause mortality, and event rates of their respective individual components using an on‐treatment approach, in patient subgroups.^a^ To describe the type and pattern of discontinuation and re‐initiation of antiplatelet drugs used in the subgroup of patients who undergo an elective PCI after the index date.
Exploratory	To analyze the associations between specific patient characteristics assessed at index date and the risk of bleeding requiring hospitalization, and of the composite endpoint of MI, stroke, and all‐cause mortality, and of their respective individual components, for patients treated with ticagrelor 60 mg using an on‐treatment approach.^a^ To describe patient characteristics at the time of MI and at the assigned index date among patients in a non‐ticagrelor cohort who are treated with a P2Y_12_ inhibitor other than ticagrelor (clopidogrel, prasugrel, or ticlopidine). To describe patient characteristics at the time of MI and at the assigned index date among patients in a non‐ticagrelor cohort who are not treated with any P2Y_12_ inhibitor. To describe event rates of bleeding outcomes (intracranial bleeding, gastrointestinal bleeding, and other bleeding requiring hospitalization, fatal bleeding, bleeding not requiring hospitalization), of CV outcomes (recurrent MI, all‐cause stroke, ischemic stroke, CV death, CHD death), all‐cause mortality, and of dyspnea and lower limb amputation in patients treated with ticagrelor 60 mg using an on‐treatment approach.^a^ To conduct sensitivity analyses (for the primary bleeding outcome and the secondary CV composite outcome) for primary objective 3, secondary objectives 1 and 2, and exploratory objective 1 using an intention‐to‐treat approach instead of an on‐treatment approach. ^a^ To investigate the associations between patient characteristics (measured at the index date) and the risk of discontinuing ticagrelor 60 mg.

Abbreviations: CHD, coronary heart disease; CV, cardiovascular; MI, myocardial infarction; PCI, percutaneous coronary intervention.

^a^ The total number of person‐years on treatment in the Primary population will be assessed across all countries. Clinical outcomes in this study will not be analyzed unless the a priori threshold of 5000 person‐years on‐treatment with ticagrelor 60 mg is met in the Primary population, as a total across all data sources.

To maximize patient inclusion and ensure a sufficient look‐back period to appropriately identify eligible patients, the study will span a broad eligibility period from the earliest date of data availability to the most recent data available at data extraction from each database. In general, the eligibility period will begin at least 3 years prior to the ticagrelor 60 mg approval date (European Union [EU]: 19 December 2015; US: 3 September 2015).

### 2.2 Patient populations

To account for differences between clinical trial selection criteria and geographic adaptations of labels for ticagrelor 60 mg, the study includes both a Primary and a Secondary population. The Primary population is defined to align as close as possible with the PEGASUS‐TIMI 54 eligible population and the US approved label.^6^ The Secondary population is defined to align as close as possible to the European approved label.^15^, ^16^, ^17^, ^18^ Both study populations will be defined in each data source. For all databases, cohort selection will begin on the US ticagrelor 60 mg approval date for the Primary population and on the EU approval date for the Secondary population. Across databases, the latest date of the cohort selection period will be 29 February, 2020.

The full eligibility criteria are described in Table 2. Briefly, the Primary population will include patients with a first prescription of ticagrelor 60 mg ≥12 months after their most recent hospitalization with a primary diagnosis of MI (i.e., their qualifying MI). The Secondary population will include patients with a first prescription of ticagrelor 60 mg, either (i) 12–24 months following their qualifying MI, or (ii) 12–36 months following their qualifying MI and with P2Y_12_ inhibitor treatment ≤12 months prior to the first ticagrelor 60 mg prescription. The date of the first ticagrelor 60 mg prescription following the qualifying MI will be defined as the index date. The exclusion criteria are based on PEGASUS‐TIMI 54 and include a history of ischemic stroke, intracranial bleeding, hepatic impairment, gastrointestinal bleeding, stage 5 chronic kidney disease (CKD), or renal failure. In recognition that the timing of ticagrelor 60 mg initiation may vary in clinical practice, the study will also include the Anytime cohort, a complementary population comprising patients with a first prescription of ticagrelor 60 mg any time after their qualifying MI.

**TABLE 2:** Study inclusion and exclusion criteria

	Primary population	Secondary population	
Inclusion criteria			
	Hospitalization with a primary diagnosis of MI during the eligibility period	Hospitalization with a primary diagnosis of MI during the eligibility period	
	–	Age ≥ 50 years at the index date	
	–	At least one of the following risk factors assessed at the index date:	
	–	Age ≥ 65 years Diabetes mellitus requiring medication (defined as ≥1 prescription any time prior to the index date) A second prior MI (defined as hospitalization with a primary diagnosis of MI in the baseline period with the date of diagnosis >30 days prior to the date of qualifying MI) Chronic non‐end‐stage renal dysfunction (defined as a diagnosis of CKD stage 1 to 4 in the baseline period)	
	A first prescription of ticagrelor 60 mg ≥12 months following their qualifying MI	A first prescription of ticagrelor 60 mg and one of the following criteria:1) 12–24 months after their qualifying MI, or 2) 24–36 months after their qualifying MI and treatment with an P2Y_12_ inhibitor ≤12 months prior to the first ticagrelor 60 mg prescription	
Exclusion criteria			
	Dies, emigrates, or disenrolls from the database (where applicable) prior to the ticagrelor 60 mg approval date Ineligibility for ticagrelor 60 mg use (restricted to the conditions possible to capture within the data sources)—one or more of the following: Concomitant use of an anticoagulant or a strong CYP3A4 inhibitor or inducer or substrate with a narrow therapeutic index (prescription within 60/90 days prior to the index date) Prior ischemic stroke, history of intracranial bleeding, severe hepatic impairment, CKD stage 5 or renal failure requiring dialysis (any time prior to the index date)		

	Gastrointestinal bleeding (within 6 months prior to the index date) <1 year of data available prior to the qualifying MI (for assessment of patient characteristics at qualifying MI^a^)		


Abbreviations: CKD, chronic kidney disease; CYP3A4, cytochrome P450 3A4; MI, myocardial infarction.

^a^ The qualifying MI is defined as the most recent hospitalization with a primary diagnosis of MI occurring prior to initiation of ticagrelor 60 mg (index date).

**FIGURE 1:**
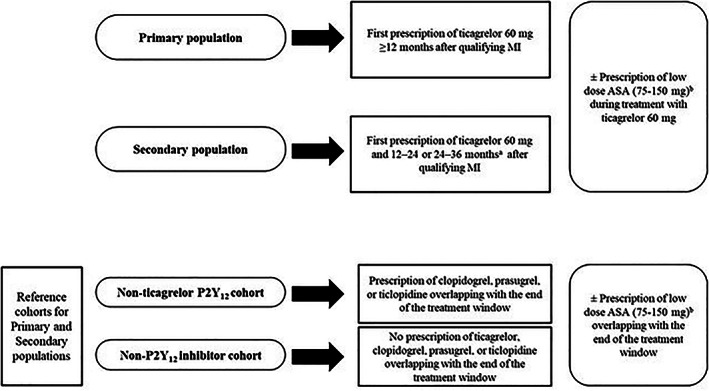
Schematic illustration of cohorts ASA, acetylsalicylic acid; MI, myocardial infarction. a Treated with a P2Y12 inhibitor (clopidogrel, prasugrel, ticagrelor 90 mg, or ticlopidine) ≤12 months prior to the ticagrelor 60 mg prescription. b ASA analyses will only be done when data available

To contextualize the characteristics of patients initiating ticagrelor 60 mg, two reference cohorts will be defined, the nonticagrelor P2Y_12_ inhibitor cohort and the non‐P2Y_12_ inhibitor cohort, applying otherwise similar eligibility criteria as for the Primary and Secondary populations. To assign reference patients into these cohorts, the median distribution of time from qualifying MI to index date in the Primary population will first be described. The rationale is to ensure that characteristics of reference patients are described at a similar time from their qualifying MI as for patients initiating ticagrelor 60 mg. Based on this median time, a treatment exposure window will be defined to categorize patients into the respective reference cohorts based on their treatment within this window. The nonticagrelor P2Y_12_ inhibitor cohort will include patients treated with clopidogrel, prasugrel, or ticlopidine at the end of the treatment window. The non‐P2Y_12_ inhibitor cohort will include patients without P2Y_12_ inhibitor treatment at the end of this window. A schematic illustration of all cohorts is presented in Figure 1.

### 2.3 Data sources

To obtain a large and geographically diverse population, data will be extracted from data sources in the US and several European countries (Germany, Italy, Sweden, and the UK). Table 3 presents detailed information on each database, along with projected numbers of patients initiating ticagrelor 60 mg ≥1 year after their most recent MI available at the time of data extraction. In general, the study will use data on inpatient and outpatient care, prescriptions, and mortality when available. An estimated total of 7250 patients prescribed ticagrelor 60 mg are expected to be included across databases.

**TABLE 3:** Description of databases in each country

Country	Database(s)	Number of unique patients and National coverage (approximate)	Projected number of patients at time of data extraction^a^
US	Optum Clinformatics® administrative claims database combines data from over 50 healthcare providers in the US (>700 hospitals and 7000 clinics). The database provides a single‐payer view of data on demographics, diagnoses, and procedures performed during outpatient visits (outpatient specialist and physician visits) or inpatient stays, and outpatient prescription records (i.e., date of dispensed prescriptions). As is typical for US commercial claims databases, comprehensive data on mortality is not available in the Optum Clinformatics database.	180 million; 55%	750
	IBM's MarketScan Commercial Claims and Medicare Supplemental database consists of administrative claims submitted from US inpatient and outpatient encounters, as well as pharmacy claims (i.e., date of dispensed prescriptions). Medical claims are linked to outpatient prescription drug claims and person‐level enrollment information. The MarketScan supplemental database profiles the healthcare experience of retirees with Medicare supplemental insurance paid by employers. The Medicare Supplemental database provides data on medical and pharmacy claims for healthcare services performed in both inpatient and outpatient settings (i.e., date of dispensed prescriptions). As is typical for US commercial claims databases, comprehensive data on mortality is not available in the MarketScan database.	203 million; 62%	1200
	Medicare health insurance database covers fee‐for‐service claims data of individuals who are aged ≥65 years, select individuals with disabilities aged <65 years, and those with end‐stage renal disease. The data cover beneficiaries' encounters with the healthcare system and receipt of therapeutic interventions, including medications (i.e., date of dispensed prescriptions), procedures, and services. Data on mortality are available.	62 million; 19%	3100
UK	CPRD GOLD contains longitudinal primary care data from over 800 general practices, whereas CRPD Aurum holds anonymized, longitudinal, primary care patient records collected from over 1100 general practices. Data includes information on patient characteristics and issued prescription medicines. HES covers data on diagnoses and procedures for all hospitalization episodes from England, whereas ONS covers information on all‐cause and cause‐specific death. Data from both HES and ONS will be linked to primary care data of the CPRD. After removal of duplicates, GOLD and Aurum primary care databases will be used for the analysis.	13.5 million; 24% (GOLD+Aurum)	250
Sweden	The National Patient Register includes information on hospital discharge diagnoses and procedures. The Swedish Prescribed Drug Register covers data on all prescribed medications purchased at Swedish community pharmacies. The Cause of Death Register includes information on date and cause of death. The registers are linked by the register holder via the unique personal identification number.	10 million; >99%	800
Germany	AOK PLUS covers longitudinal claims data submitted from primary, secondary, and tertiary care, and contains information on patient demographics, inpatient hospitalizations, outpatient visits, procedures, mortality (inpatient and outpatient date of death), and outpatient prescriptions dispensed in pharmacies.	3.5 million; 4%	150
Italy	ReS database includes data on patient demographics, pharmacy‐dispensed prescription drugs, inpatient diagnoses and procedures, and date of in‐hospital mortality. For deaths in the outpatient setting, neither date of death nor cause of death is recorded in ReS.	5.5 million; 10%	1000
**Total estimated number of patients included across databases** ^a^			**7250**

Abbreviations: AOK, Allgemeine Ortskrankenkasse; CPRD, Clinical Practice Research Datalink; HES, hospital episode statistics; IBM, International Business Machines; MI, myocardial infarction; ONS, Office for National Statistics; ReS, Ricerca e Salute; Italian Research and Health Foundation; UK, United Kingdom; US, United States.

^a^ Projected approximate numbers of patients initiating ticagrelor 60 mg ≥1 year after their most recent MI, expected to be available in the data sources at the time of data extraction.

### 2.4 Definition of treatment exposure and persistence

Treatment persistence for ticagrelor 60 mg will be defined as time on continuous treatment. To account for potential stockpiling, overlapping prescriptions will be assumed to be used sequentially. The treatment episode will be considered as continuous if the gap between days with available medication supply (grace period) is less than two times the number of days' supply of the most recent prescription. Patients with no further evidence of treatment before the end of the grace period will be defined as having discontinued treatment. The discontinuation date is defined as the date of the last prescription plus the number of days' supply in that last prescription plus 7 days, or the date of switch, or the date of death, whichever occurs first.

### 2.5 Clinical outcomes

Study measures, including outcomes, are defined using diagnosis, procedure, and medication codes in the formats used in each database; full definitions are presented in Table S1.

To ensure sufficient precision, clinical outcomes for all study populations will only be analyzed if the a priori threshold of 5000 person‐years on‐treatment with ticagrelor 60 mg is met in the Primary population, as a total across all data sources.

The primary outcome, bleeding requiring hospitalization, is defined as an inpatient admission with at least one overnight stay with a primary diagnosis of bleeding. In a sensitivity analysis, the primary bleeding outcome is defined as bleeding events associated with an inpatient admission of ≥2 overnight stays. Additional bleeding outcomes include the individual components of the primary outcome (hospitalization for intracranial hemorrhage, gastrointestinal bleeding and other bleeding, respectively), bleeding episodes not requiring hospitalization, and fatal bleeding.

Outcomes were identified on the event date recorded in the EHD; for composite outcomes, the earliest date of any of the components was used to define the event date. The secondary CV composite outcome is defined as the composite of hospitalization for MI or stroke, and all‐cause mortality. Additional outcomes include the individual components of the secondary CV composite outcome, three‐point MACE (composite of hospitalization for MI or stroke, and CV death), hospitalization for ischemic stroke, CV death, as well as coronary heart disease (CHD) death. To ensure harmonized definitions across all databases, fatal bleeding, CHD death, and CV disease (CVD) death are defined as death within 28 days of an inpatient hospital admission with a primary diagnosis of bleeding, CHD, and CVD, respectively. Sensitivity analyses for these outcomes will be performed in databases reporting cause of death from death certificates (UK and Sweden). Exploratory outcomes include dyspnea and lower‐limb amputation, as well as bleeding requiring transfusion (only available in the US databases).

Due to insufficiently detailed clinical data in all databases to adequately adjust for imbalances in clinical characteristics between cohorts for comparative analyses, clinical outcomes will only be analyzed in the ticagrelor 60 mg cohorts (i.e., not in the reference cohorts).

**FIGURE 2:**
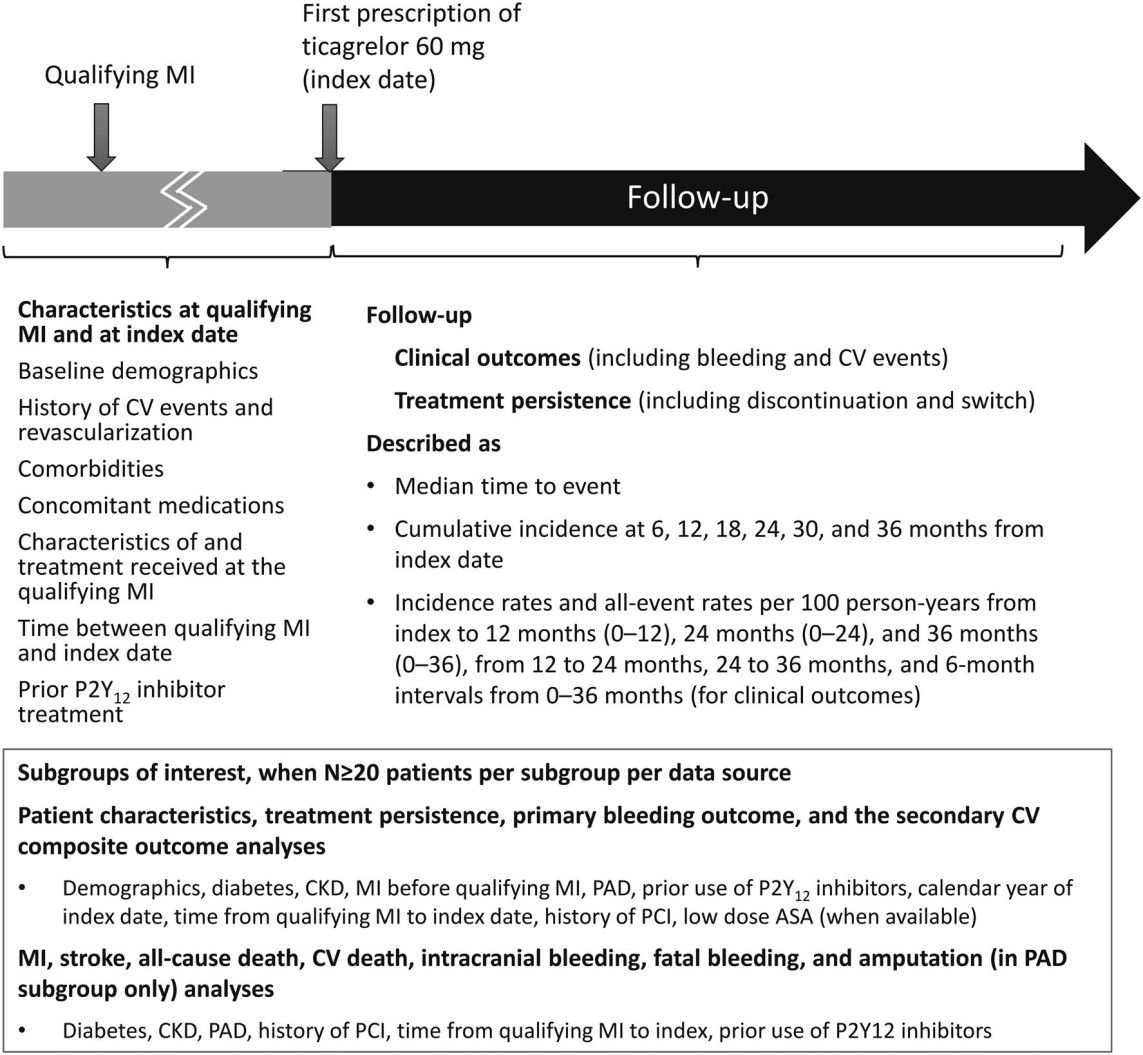
Study design ASA, acetylsalicylic acid; CKD, chronic kidney disease; CV, cardiovascular; MI, myocardial infarction; PAD, peripheral artery disease; PCI, percutaneous coronary intervention

Subgroup analyses of interest are described in Figure 2.

### 2.6 Study organizational structure

The study will be conducted by the sponsor (AstraZeneca) in collaboration with a contract research organization (Evidera), encompassing both cardiology and epidemiology expertise, with oversight from a scientific committee of cardiology experts external to both companies. The external scientific committee is responsible for: (i) The planning, development and scientific integrity of all publications and presentations derived from the ALETHEIA study in collaboration with AstraZeneca; (ii) The content and implementation of the study protocol and analysis plan, its interpretation, and reporting of the study results. All authors take responsibility for the veracity of the data. Full details of the study organization are provided in Table S2.

### 2.7 Statistical analyses

Patient characteristics (as outlined in Figure 2) will be summarized at qualifying MI and at index date for patients treated with ticagrelor 60 mg, for the nonticagrelor P2Y_12_ inhibitor cohort, and for the non‐P2Y_12_ inhibitor cohort using descriptive statistics. All other statistical analyses will be performed among patients treated with ticagrelor 60 mg.

Persistence to treatment with ticagrelor 60 mg, including median time until discontinuation (if and when reached during follow‐up), and the percentage of patients remaining on treatment at specific timepoints from index date (Figure 2), will be calculated using the Kaplan–Meier (KM) method. Additionally, switches to another P2Y_12_ inhibitor or an anticoagulant will be similarly calculated.

The KM method will be used for calculating median times to clinical outcome events (if and when reached during follow‐up) and cumulative incidence at specific time points (Figure 2), presented along with 95% confidence intervals (CI). Incidence rates per 100 person‐years along with 95% CI will be calculated as the total number of patients with the event during follow‐up divided by the total person‐time at risk; during the intervals outlined in Figure 2. For events that can occur more than once, the all‐event rates (i.e., wherein patients are not censored at the occurrence of the event of interest) will also be calculated within the same intervals. Diagnoses for the same type of event recorded within 30 days will be assumed to be for follow‐up care, rather than a new event. For all outcomes, only timepoints at which a minimum of five events are recorded will be described.

Cox proportional hazards regression models will be used to assess the association of baseline patient characteristics (measured at index date) with the risk of the primary bleeding outcome, with the secondary CV composite outcome and with treatment discontinuation, respectively. Hazard ratios (HR) and 95% CIs will be presented.

The primary outcomes analyses will be based on an on‐treatment approach, censoring at treatment discontinuation. Additionally, an intention‐to‐treat approach will be used as a sensitivity analysis for the primary bleeding outcome and the secondary CV composite outcome. Sensitivity analyses will also be performed using two alternative definitions of treatment discontinuation, where the grace period is defined as 60 days in all databases, or where the discontinuation date is defined as the date of the last prescription of ticagrelor 60 mg plus half the number of days' supply in that last prescription plus 7 days.

Meta‐analyses will be applied to combine estimates derived from each country/database. Each meta‐analysis will be preceded by an assessment of heterogeneity in terms of patient characteristics and estimates. Heterogeneity will be quantified by the *I* ^2^‐statistic,^19^ and reasons for any observed heterogeneity will be explored prior to estimating the components of the meta‐analysis. A fixed‐effects approach will be used as the main method; random‐effects analyses will also be performed. The meta‐analysis results will be presented in Forest plots displaying the individual database‐level estimates with corresponding 95% CIs.

Regression models combined in the meta‐analyses will use predefined sets of covariates. These covariates will be selected based on the results of the country‐level regression models and clinical knowledge.

### 2.8 Sample size estimation

No power calculations were performed as all objectives are descriptive in nature. The a priori threshold of 5000 person‐years on‐treatment for outcomes analyses among patients treated with ticagrelor 60 mg was based on an assumed event rate for bleeding requiring hospitalization of 0.5 per 100 person‐years (the annualized major bleeding rate was approximately 0.9% in the PEGASUS‐TIMI 54 trial).^17^ With a sample size of 5000 patients (assuming an average of 1 year on treatment), the 95% CI would be 0.3–0.7 events per 100 person‐years. The event rate for MACE (hospitalization for MI, stroke, or CV death) was expected to be approximately 2.5 per 100 person‐years (the annualized rate for this outcome was approximately 2.7% in the PEGASUS‐TIMI 54 trial).^17^, ^20^ Given that this outcome is expected to occur more frequently than bleeding requiring hospitalization, the threshold of 5000 person‐years will be applied for all clinical outcome analyses.

### 2.9 Ethical considerations

This observational study will be performed in accordance with ethical principles consistent with the Declaration of Helsinki and the International Society for Pharmacoepidemiology guidelines on Good Pharmacoepidemiology Practice. The final protocol has been approved by Ethics Committees/Institutional Review Boards, as applicable in each of the countries.

This study does not involve any primary data collection; hence, subject informed consent is not applicable.

## 3 DISCUSSION

ALETHEIA aims to enhance the understanding of patient characteristics, treatment patterns, and bleeding and CV outcomes in patients with a history of MI on extended DAPT with ticagrelor 60 mg in routine clinical practice. The study will include patient‐level data from seven observational EHD sources from five countries across Europe and the US, to obtain a large and geographically diverse patient sample. The main study populations are defined to align, as much as possible in observational EHD, with the overall PEGASUS‐TIMI 54 population (the Primary population)^6^ and its subgroup relevant to geographic adaptations of the ticagrelor 60 mg label (the Secondary population, aligned with the European label).^17^, ^18^ Given the lack of robust data on ticagrelor 60 mg use in routine clinical practice, ALETHEIA is therefore designed to supplement and further explore clinical trial findings by generating information that may be used to guide appropriate patient selection for this therapy.

The stepwise design with an a priori threshold of person‐years on treatment has been implemented to ensure that precise estimates of clinical outcomes can be calculated, to more accurately inform clinical decision‐making.

Data from routine clinical practice on prescription patterns and clinical outcomes in patients treated with ticagrelor 60 mg post‐MI are very limited. In a recent observational study in 181 patients in Italy, including those with a previous MI treated with ticagrelor 60 mg twice daily plus aspirin, the 1‐year risk of MACE was 5.0%, with MI being the main contributor (3.9%), while no major bleeding events occurred.^11^

Based on the TWILIGHT trial^21^ findings and latest updates to clinical guidelines,^8^ monotherapy with a P2Y_12_ inhibitor is an emerging treatment strategy for patients post‐percutaneous coronary intervention. In this study, all patients are assumed to be on concomitant ASA, since data on medications that can be purchased without a prescription, such as ASA, are not available in all countries. Based on the product labels and the time period in which the observations the ALETHEIA study took place (most patients pre‐2019), concomitant use of ASA is a reasonable assumption. The extent of concomitant ASA use will be described in databases capturing this data, to assess consistency with this assumption.

One strength of this study is that patient characteristics will also be described among patients treated with a nonticagrelor P2Y_12_ inhibitor and those who are not treated with any P2Y_12_ inhibitor, at a comparable time from their MI to patients initiating ticagrelor 60 mg. This will provide useful contextualization of the findings, as patient characteristics (e.g., risk factors, disease severity, comedications), prescriber preferences, and local guidelines may influence the selection of treatments prescribed in clinical practice. The main reasons for not performing comparative outcomes analyses include the expected lack of complete information on all covariates assumed to be needed to adequately balance the populations for baseline risk, leading to potential residual confounding, as well as key methodological challenges for comparative analyses, such as determining use of low‐dose ASA, which is not captured in all databases. While the data are physician‐reported and the claims data are adjudicated for reimbursement purposes, no additional adjudication of clinical events is performed, a limitation shared with most observational database studies. However, some databases used in the study (e.g., the Swedish National Inpatient Register) have been validated for clinical diagnoses, including CV disease.^22^, ^23^

The study also has a number of limitations typical of multi‐country, register‐based, observational studies. First, while the data sources used in this study reflect routine clinical practice, they are not primarily established for research purposes. Therefore, coding practices of diagnoses and medications (e.g., prescriptions issued vs. filled) may vary between databases. While study definitions have been harmonized across databases and based on validated algorithms wherever possible,^22^ these differences may influence the ascertainment of patients for inclusion in the study and of clinical outcomes. Second, characteristics and baseline risks may differ between countries due to differences in patient characteristics, healthcare systems, prescribing practices, and other population‐level factors. This may limit our ability to conduct meta‐analyses, as well as the generalizability of the study findings, but this limitation is offset by the advantages of using multiple databases, which includes increased geographical representativeness and sample size. In this study, analyses are harmonized using common definitions across the databases. Additionally, sensitivity analyses (e.g., around prescription length) and the calculation of the *I* ^2^‐statistic for meta‐analyses intends to mitigate and quantify heterogeneity across databases. While outcomes based on cause of death will be defined using the same definition across all databases to ensure harmonization, the contextualization of these findings with sensitivity analyses using data from death certificates (available from UK and Sweden) will be a valuable contribution in advancing methodology in observational studies. Third, the European databases included in this study do not contain comprehensive information on blood transfusions, so major bleeding events are primarily defined using only inpatient diagnostic codes for bleeding. Still, an exploratory outcome on bleeding requiring transfusions will be assessed in the US databases, where transfusion procedures are expected to be more reliably captured. Furthermore, common bleeding definitions used in randomized trials require laboratory parameters to categorize bleeding events, but as this information is not consistently available from EHD sources, bleeding scales were not used in in ALETHEIA. Since comprehensive information on mortality is available in US Medicare claims but not in Optum Clinformatics and IBM MarketScan, additional meta‐analyses of outcomes, whether or not these outcomes include death, will be performed as a sensitivity analysis in a restricted study population, excluding patients from the Optum and MarketScan databases. The rationale is to provide results for both the bleeding and CV outcomes in populations with similar age distributions.

Fourth, the 5000 person‐years on treatment threshold is considered as the minimum number required for sufficient precision in clinical outcome assessment. While this threshold is expected to be met, person‐years on treatment is a function of both time on treatment and number of patients, and is thereby influenced by the databases' available duration of longitudinal data as well as their national coverage.

Fifth, to allow contextualization of patient characteristics, reference cohorts of nonticagrelor patients will be described at a similar time point from their qualifying MI as will be observed among the patients initiating ticagrelor 60 mg. In routine clinical practice, patients treated for ACS may continue being prescribed a P2Y_12_ inhibitor more than 12 months after an MI, despite not being intended for extended antiplatelet treatment. To limit the inclusion of such patients in the nonticagrelor P2Y_12_ inhibitor cohort, categorization of patients into either reference cohort will be based on their treatment at a minimum of 15 months after their qualifying MI. Exposure to nonticagrelor P2Y_12_ inhibitors or lack of exposure to P2Y_12_ inhibitors may therefore be assessed after the index date for some patients, introducing a survival bias to patients in the reference cohort. Although the extent of this bias cannot be quantified a priori, the impact is likely minimal given the short maximum timeframe between the index date (≥12 months post‐MI) and the time of treatment assessment (≥15 months post‐MI). Sixth, while the primary analyses will be conducted using an on‐treatment approach, reflecting events that occur when patients are receiving ticagrelor 60 mg, censoring at treatment discontinuation may introduce bias due to informative censoring. This could occur if the risk of discontinuing treatment is related to the events of interest, thus violating the key assumption in survival analyses that censoring events are random. While difficult to properly account for, the likelihood of such bias being introduced will be assessed by investigating baseline factors associated with discontinuation. To further assess the potential risk of informative bias, event rates will also be calculated using an intention‐to‐treat approach.

In conclusion, despite the increasing recognition of observational studies to inform healthcare decision‐making, knowledge about treatment patterns and associated outcomes in patients treated with ticagrelor 60 mg in routine clinical practice is limited. The multi‐country, observational ALETHEIA study is designed to address this gap. The study objectives and the a priori specified stepwise approach used for outcomes analyses are expected to generate useful insights for clinical decision‐making and treatment optimization.

## CONFLICT OF INTEREST

The ALETHEIA study was sponsored by AstraZeneca. Eva Lesén, Christopher Hewitt, and Jonatan Hedberg are employees of AstraZeneca. Jason Simeone and Dimitra Lambrelli are employees of Evidera, which received funding from AstraZeneca for the conduct of this study. Marc Bonaca reports institutional grant support from Amgen, Arca, AstraZeneca, Bayer, Janssen, Merck, Novo Nordisk, Pfizer, Sanofi, WraSer. Aldo P Maggioni reports consultancy fees from AstraZeneca, Bayer, Fresenius, and Novartis. Albert Ariza‐Solé reports consultancy fees from AstraZeneca. Evangelos Giannitsis reports institutional grants from Roche Diagnostics and Daiichi Sankyo, and consultancy fees from AstraZeneca, Bayer Vital, Boehringer Ingelheim, BRAHMS Deutschland, Daiichi Sankyo, Idorsia, Mitsubishi Chemicals Novo Nordisk, Radiometer, and Roche Diagnostics. Tomas Jernberg reports institutional grants from Novartis and consultancy fees from AstraZeneca, Bayer, Novartis and Sanofi. Jurrien ten Berg reports institutional research grants from ZonMw and AstraZeneca, and consultancy fees from Accu‐Metrics, AstraZeneca, Bayer, Boehringer Ingelheim, Bristol Myers Squibb, Daiichi Sankyo, Eli Lilly, Ferrer, Idorsia, Pfizer, and The Medicines Company. Robert F Storey reports institutional research grants/support from AstraZeneca, Cytosorbents, GlyCardial Diagnostics and Thromboserin; consultancy fees from Amgen, AstraZeneca, Bayer, Bristol Myers Squibb/Pfizer, Cytosorbents, GlyCardial Diagnostics, Haemonetics, Hengrui, Idorsia, PhaseBio, Portola, Sanofi Aventis and Thromboserin; and honoraria from AstraZeneca, Bayer, Bristol Myers Squibb/Pfizer, Intas Pharmaceuticals and Medscape. The authors declare no other potential conflicts of interest.

## Supporting information


**Appendix S1:** Supporting Information

## Data Availability

Data sharing not applicable as no data were generated or analysed during the current study.

## References

[1] Khan MA , Hashim MJ , Mustafa H , et al. Global epidemiology of ischemic heart disease: results from the global burden of disease study. Cureus. 2020;12(7):e9349. 32742886 10.7759/cureus.9349 PMC7384703

[2] Jernberg T , Hasvold P , Henriksson M , Hjelm H , Thuresson M , Janzon M . Cardiovascular risk in post‐myocardial infarction patients: Nationwide real world data demonstrate the importance of a long‐term perspective. Eur Heart J. 2015;36(19):1163‐1170. 25586123 10.1093/eurheartj/ehu505

[3] Gremmel T , Michelson AD , Frelinger AL III , Bhatt DL . Novel aspects of antiplatelet therapy in cardiovascular disease. Res Pract Thromb Haemost. 2018;2(3):439‐449. 30046748 10.1002/rth2.12115 PMC6046593

[4] Koski R , Kennedy B . Comparative review of oral P2Y12 inhibitors. P T. 2018;43(6):352‐357. 29896034 PMC5969212

[5] Mauri L , Kereiakes DJ , Yeh RW , et al. Twelve or 30 months of dual antiplatelet therapy after drug‐eluting stents. N Engl J Med. 2014;371(23):2155‐2166. 25399658 10.1056/NEJMoa1409312 PMC4481318

[6] Bonaca MP , Bhatt DL , Cohen M , et al. Long‐term use of ticagrelor in patients with prior myocardial infarction. N Engl J Med. 2015;372(19):1791‐1800. 25773268 10.1056/NEJMoa1500857

[7] Fanari Z , Malodiya A , Weiss SA , Hammami S , Kolm P , Weintraub WS . Long‐term use of dual antiplatelet therapy for the secondary prevention of atherothrombotic events: meta‐analysis of randomized controlled trials. Cardiovasc Revasc Med. 2017;18(1):10‐15. 10.1016/j.carrev.2016.07.006 PMC5250594 27477306

[8] Knuuti J , Wijns W , Saraste A , et al. 2019 ESC guidelines for the diagnosis and management of chronic coronary syndromes. Eur Heart J. 2020;41(3):407‐477. 31504439 10.1093/eurheartj/ehz425

[9] Levine GN , Bates ER , Bittl JA , et al. ACC/AHA guideline focused update on duration of dual antiplatelet therapy in patients with coronary artery disease: a report of the American College of Cardiology/American Heart Association task force on clinical practice guidelines: an update of the 2011 ACCF/AHA/SCAI guideline for percutaneous coronary intervention, 2011 ACCF/AHA guideline for coronary artery bypass graft surgery, 2012 ACC/AHA/ACP/AATS/PCNA/SCAI/STS guideline for the diagnosis and Management of Patients with Stable Ischemic Heart Disease, 2013 ACCF/AHA guideline for the management of ST‐elevation myocardial infarction, 2014 AHA/ACC guideline for the Management of Patients with non‐ST‐elevation acute coronary syndromes, and 2014 ACC/AHA guideline on perioperative cardiovascular evaluation and Management of Patients Undergoing Noncardiac Surgery. Circulation. 2016;134(10):e123‐e155. 27026020 10.1161/CIR.0000000000000404

[10] Valgimigli M , Bueno H , Byrne RA , et al. 2017 ESC focused update on dual antiplatelet therapy in coronary artery disease developed in collaboration with EACTS: the task force for dual antiplatelet therapy in coronary artery disease of the European Society of Cardiology (ESC) and of the European Association for Cardio‐Thoracic Surgery (EACTS). Eur Heart J. 2018;39(3):213‐260. 28886622 10.1093/eurheartj/ehx419

[11] Cesaro A , Taglialatela V , Gragnano F , et al. Low‐dose ticagrelor in patients with high ischemic risk and previous myocardial infarction: a multicenter prospective real‐world observational study. J Cardiovasc Pharmacol. 2020;76(2):173‐180. 32569017 10.1097/FJC.0000000000000856

[12] Katkade VB , Sanders KN , Zou KH . Real world data: an opportunity to supplement existing evidence for the use of long‐established medicines in health care decision making. J Multidiscip Healthc. 2018;11:295‐304. 29997436 10.2147/JMDH.S160029 PMC6033114

[13] US FDA . Framework for FDA's Real‐World Evidence Program. 2018. https://www.fda.gov/media/120060/download.

[14] Eichler HG , Koenig F , Arlett P , et al. Are novel, nonrandomized analytic methods fit for decision making? The need for prospective, controlled, and transparent validation. Clin Pharmacol Ther. 2020;107(4):773‐779. 31574163 10.1002/cpt.1638 PMC7158212

[15] Dellborg M , Bonaca MP , Storey RF , et al. Efficacy and safety with ticagrelor in patients with prior myocardial infarction in the approved European label: insights from PEGASUS‐TIMI 54. Eur Heart J Cardiovasc Pharmacother. 2019;5(4):200‐206. 31218354 10.1093/ehjcvp/pvz020 PMC6749839

[16] Ticagrelor [SmPC]. Brilique 60 mg: Summary of product characteristics. 2019.

[17] Bonaca MP , Bhatt DL , Steg PG , et al. Ischaemic risk and efficacy of ticagrelor in relation to time from P2Y12 inhibitor withdrawal in patients with prior myocardial infarction: insights from PEGASUS‐TIMI 54. Eur Heart J. 2016;37(14):1133‐1142. 26491109 10.1093/eurheartj/ehv531

[18] Bonaca MP , Storey RF , Theroux P , et al. Efficacy and safety of ticagrelor over time in patients with prior MI in PEGASUS‐TIMI 54. J Am Coll Cardiol. 2017;70(11):1368‐1375. 28882235 10.1016/j.jacc.2017.07.768

[19] Higgins JP , Thompson SG . Quantifying heterogeneity in a meta‐analysis. Stat Med. 2002;21(11):1539‐1558. 12111919 10.1002/sim.1186

[20] Bonaca MP , Bhatt DL , Oude Ophuis T , et al. Long‐term tolerability of ticagrelor for the secondary prevention of major adverse cardiovascular events: a secondary analysis of the PEGASUS‐TIMI 54 trial. JAMA Cardiol. 2016;1(4):425‐432. 27438319 10.1001/jamacardio.2016.1017

[21] Mehran R , Baber U , Sharma SK , et al. Ticagrelor with or without aspirin in high‐risk patients after PCI. N Engl J Med. 2019;381(21):2032‐2042. 31556978 10.1056/NEJMoa1908419

[22] Friberg L , Skeppholm M . Usefulness of health registers for detection of bleeding events in outcome studies. Thromb Haemost. 2016;116(6):1131‐1139. 27617328 10.1160/TH16-05-0400

[23] Ludvigsson JF , Andersson E , Ekbom A , et al. External review and validation of the Swedish national inpatient register. BMC Public Health. 2011;11:450. 21658213 10.1186/1471-2458-11-450 PMC3142234

