# Supplementary material for: Extended dual antiplatelet therapy with ticagrelor 60 mg in patients with prior myocardial infarction: The design of ALETHEIA, a multi‐country observational study
Source: Clin Cardiol. 2021 Aug 8;44(10):1333–43. doi: 10.1002/clc.23702 (PMC8495086; doi:10.1002/clc.23702)
Supplement: Supplementary file 1 — Appendix S1: Supporting Information [file CLC-44-1333-s001.docx]

# SUPPLEMENTARY DATA

**Supplementary Table 1. Definitions/Codes for outcomes, diagnoses, procedures, and medications**

| **OUTCOMES** | |
| --- | --- |
| Bleeding requiring hospitalization | Overnight hospitalization for intracranial, gastrointestinal, and other bleeding, as defined below |
| Intracranial bleeding requiring hospitalization | Overnight hospitalization with an ICD code in primary position for intracranial bleeding: ICD-10: I60-I62, S06.4-S06.6; ICD-9: 430- 432, 852 |
| Gastrointestinal bleeding requiring hospitalization | Overnight hospitalization with an ICD code in primary position for gastrointestinal bleeding:  ICD-10: K22.1, K22.6, K25-K28 (only subcodes .0, .2, .4, .6), K29.0, K62.5, K66.1, K92.0-K92.2, I85.0, I93.8;  ICD-9: 530.7, 531-534 (only subcodes .0, .2, .4, .6) 569.3, 578.0, 578.1, 578.9, 456.0 |
| Other bleeding (bleeding other than intracranial hemorrhage or gastrointestinal bleeding) requiring hospitalization | Overnight hospitalization with an ICD code in primary position for other bleeding:  ICD-10: H11.3, H31.3, H35.6, H43.1, H45.0, H92.2, I31.2, J94.2, M25.0, N93.9, N95.0, N501A, T81.0, D50.0, D62.9, R04, R31, R58, G95.1, S27.1, S27.2, S26.0; ICD-9: 246.3, 336.1, 363.6, 423.0, 459.0, 596.7, 599.7, 626.6, 626.8, 626.9, 627.1, 719.1, 770.3, 784.7, 784.8, 786.3, 860.2-860.5 |
| Fatal bleeding | Death where the primary cause is registered with a diagnosis for intracranial, gastrointestinal or other bleeding |
| Bleeding not requiring hospitalization | An ICD code for intracranial, gastrointestinal, or other bleeding during an outpatient or emergency care visit or an inpatient episode without an overnight stay (e.g., day case) |
| **CV outcomes** (all codes refer to inpatient diagnoses in primary position, unless stated otherwise) | |
| Secondary CV composite outcome | Composite of hospitalization for MI or stroke, and all-cause mortality |
| MI | ICD-10: I21-I22; ICD-9: 410 |
| Stroke | ICD-10: I60-I61, I63-I64; ICD-9: 430-431, 433-434 |
| All-cause mortality | Death from any cause |
| Ischemic stroke | ICD-10: I63-I64; ICD-9: 433-434 |
| CV death | ICD-10: Death with a primary cause in the I chapter; ICD-9: 390-459 |
| CHD death | Death with an ICD code for CHD:  ICD-10: I21-I23, I46.1, I46.9, Y84.0; ICD-9: 410, 427.5, 429.5-7, E879, V12.53  OR death within 30 days following a PCI^a^ or CABG^b^ procedure (codes defined below) |
| Three-point MACE | Composite of hospitalization for MI or stroke, and cardiovascular death |
| **Exploratory outcomes** | |
| Dyspnea | ICD-10: R06.0 |
| Amputation | Procedure code for lower limb amputation^c^ |
| **PATIENT CHARACTERISTICS** | |
| **Prior history of CV events** (inpatient codes in primary position) | |
| MI hospitalization | ICD-10: I21-I23; ICD-9: 410, 429.7 |
| Angina pectoris | Stable: ICD-10: I20.1, I20.8, I20.9, I24.9; ICD-9: 413.0, 413.1, 413.9  Unstable: ICD-10: I20.0; ICD-9: 411.1 |
| **Major comorbidities** (inpatient or specialist outpatient codes in any position, unless stated otherwise) ^21^ | |
| *CV comorbidities* |  |
| Diabetes | Prescription of an antidiabetic medication (insulin and other glucose-lowering drugs) |
| Chronic non-end stage renal dysfunction | ICD-10: N18.1-N18.4, N18.9, N19; ICD-9: 585.1-585.4, 585.9, 586 |
| Peripheral arterial disease | ICD-10: I70, 173.9, I74, N28.0, K55.0-K55.1, E10.5, E11.5, E12.5, E13.5, E14.5  ICD-9: 440, 443.9, 557.0, 557.1 |
| Hypertension | ICD-10: I10-I13, I15; ICD-9: 401-405 |
| Dyslipidemia | ICD-10: E78.0-E78.5; ICD-9: 272.0-272.4 |
| Heart failure | ICD-10: I11.0, I13.0, I13.2, I50, I97.1; ICD-9: 428 |
| Atrial fibrillation | ICD-10: I48; ICD-9: 427.3 |
| *Other comorbidities* |  |
| Obesity | ICD-10: E66; ICD-9: 278.0 |
| COPD or asthma | ICD-10: J40, J45, J47; ICD-9: 491-493, 494.0-494.1, 496; Prescription of orciprenaline; salbutamol; terbutaline; fenoterol; rimiterol; hexoprenaline; pirbuterol; tretoquinol; tulobuterol; salmeterol; formoterol; reproterol; procaterol; bitolterol; indacaterol; olodaterol; salbutamol and sodium cromoglicate; reproterol and sodium cromoglicate; salmeterol and fluticasone; formoterol and budesonide; formoterol and beclomethasone; formoterol and mometasone; vilanterol and fluticasone furoate; formoterol and fluticasone; salmeterol and budesonide; salbutamol and beclometasone; indacaterol and mometasone; fenoterol and ipratropium bromide; albuterol + ipratropium; salbutamol and ipratropium bromide; umeclidinium + vilanterol; vilanterol and umeclidinium bromide; indacaterol + glycopyrronium; formoterol and aclidinium bromide; olodaterol and tiotropium bromide; formoterol and glycopyrronium bromide; vilanterol, umeclidinium bromide and fluticasone furoate; formoterol, glycopyrronium bromide and beclometasone; formoterol and tiotropium bromide; formoterol, glycopyrronium bromide and budesonide; indacaterol, glycopyrronium bromide and mometasone; beclomethasone; budesonide; flunisolide; betamethasone; fluticasone; triamcinolone; mometasone; ciclesonide; fluticasone furoate; ipratropium; oxitropium bromide; stramoni preparations; tiotropium bromide; aclidinium bromide; glycopyrronium; umeclidinium bromide; revefenacin; tiotropium bromide, combinations; cromoglicic acid; nedocromil; fenspiride; ephedrine; methoxyphenamine; orciprenaline, combinations; carbuterol; bambuterol; clenbuterol; terbutaline, combinations; clenbuterol and ambroxol; dyphylline (AKA diprophylline); diprophylline; choline theophyllinate; proxyphylline; theophylline; aminophylline; etamiphylline; theobromine; bamifylline; acefylline piperazine; bufylline; doxofylline; mepyramine theophyllinacetate; combinations of xanthines; diprophylline, combinations; theophylline, combinations excl. psycholeptics; aminophylline, combinations; theobromine, combinations; theophylline, combinations with psycholeptics; diprophylline and adrenergics; choline theophyllinate and adrenergics; proxyphylline and adrenergics; theophylline and adrenergics; aminophylline and adrenergics; etamiphylline and adrenergics; zafirlukast; pranlukast; montelukast; ibudilast; montelukast, combinations; eprozinol; omalizumab; seratrodast; roflumilast; reslizumab; mepolizumab; benralizumab; potassium iodide |
| Anemia | ICD-10: D50, D62, D64.9; ICD-9: 280, 285.1, 285.8-285.9 |
| Prior history of bleeding requiring hospitalization | Any of the diagnoses listed above under “Bleeding requiring hospitalization” in primary or secondary position |
| Hepatic disease | ICD-10: B18, K70.0-K70.3, K70.9, K71.3-K71.5, K71.7, K73-K74, K76.0, K76.2-K76.4, K76.8, K76.9, Z94.4; ICD-9: 070, 570, 571, 573, V42.7 |
| Malignant neoplasms | ICD-10 C chapter: C00-C97; ICD-9: 140-209 |
| *Characteristics of and treatment received at the qualifying MI* |  |
| PCI | PCI^a^, procedures |
| CABG | CABG^b^ procedures |
| *Prior antiplatelet treatment* |  |
| ASA (excluding US) | Acetylsalicylic acid 75-150 mg; ASA combinations with PPI; simvastatin and acetylsalicylic acid; pravastatin and acetylsalicylic acid; simvastatin, acetylsalicylic acid and ramipril; rosuvastatin and acetylsalicylic acid; atorvastatin, acetylsalicylic acid and ramipril; atorvastatin and acetylsalicylic acid; atorvastatin, acetylsalicylic acid and perindopril; sotalol and acetylsalicylic acid; metoprolol and acetylsalicylic acid; bisoprolol and acetylsalicylic acid; sotalol and acetylsalicylic acid; metoprolol and acetylsalicylic acid; bisoprolol and acetylsalicylic acid |
| P2Y_12_ inhibitors | Ticagrelor; clopidogrel; prasugrel; ticlopidine |
| *Comedications* |  |
| ACE inhibitors | Captopril; enalapril; lisinopril; perindopril; ramipril; quinapril; benazepril; cilazapril; fosinopril; trandolapril; spirapril; delapril; moexipril; temocapril; zofenopril; imidapril; captopril and diuretics; enalapril and diuretics; lisinopril and diuretics; perindopril and diuretics; ramipril and diuretics; quinapril and diuretics; benazepril and diuretics; cilazapril and diuretics; fosinopril and diuretics; delapril and diuretics; moexipril and diuretics; zofenopril and diuretics; enalapril and lercanidipine; lisinopril and amlodipine; perindopril and amlodipine; ramipril and felodipine; enalapril and nitrendipine; ramipril and amlodipine; trandolapril and verapamil; delapril and manidipine; perindopril, amlodipine and indapamide; perindopril and bisoprolol; ramipril, amlodipine and hydrochlorothiazide; perindopril, bisoprolol and amlodipine; rosuvastatin, amlodipine and lisinopril; atorvastatin, amlodipine and perindopril; atorvastatin, acetylsalicylic acid and perindopril; rosuvastatin, perindopril and indapamide; rosuvastatin, amlodipine and perindopril; atorvastatin and perindopril; simvastatin, acetylsalicylic acid and ramipril; atorvastatin, acetylsalicylic acid and ramipril; rosuvastatin and ramipril |
| ARB | Losartan; eprosartan; valsartan; irbesartan; tasosartan; candesartan; telmisartan; olmesartan medoxomil; azilsartan medoxomil; fimasartan; losartan and diuretics; eprosartan and diuretics; valsartan and diuretics; irbesartan and diuretics; candesartan and diuretics; telmisartan and diuretics; olmesartan medoxomil and diuretics; azilsartan medoxomil and diuretics; fimasartan and diuretics; valsartan and amlodipine; olmesartan medoxomil and amlodipine; telmisartan and amlodipine; irbesartan and amlodipine; losartan and amlodipine; candesartan and amlodipine; valsartan and lercanidipine; fimasartan and amlodipine; valsartan, amlodipine and hydrochlorothiazide; valsartan and aliskiren; olmesartan medoxomil, amlodipine and hydrochlorothiazide; valsartan and sacubitril; valsartan and nebivolol; candesartan, amlodipine and hydrochlorothiazide; irbesartan, amlodipine and hydrochlorothiazide; rosuvastatin and valsartan |
| ARNI | Valsartan and sacubitril |
| MRA | Spironolactone; potassium canrenoate; eplerenone; canrenone |
| β-blockers | Alprenolol; oxprenolol; pindolol; propranolol; timolol; sotalol; nadolol; mepindolol; carteolol; tertatolol; bopindolol; bupranolol; penbutolol; cloranolol; practolol; metoprolol; atenolol; acebutolol; betaxolol; bevantolol; bisoprolol; celiprolol; esmolol; epanolol; s-atenolol; nebivolol; talinolol; landiolol; labetalol; carvedilol; oxprenolol and thiazides; propranolol and thiazides; timolol and thiazides; sotalol and thiazides; nadolol and thiazides; metipranolol and thiazides, combinations; metoprolol and thiazides; atenolol and thiazides; acebutolol and thiazides; bevantolol and thiazides; bisoprolol and thiazides; nebivolol and thiazides; metoprolol and thiazides, combinations; labetalol and thiazides; oxprenolol and other diuretics; pindolol and other diuretics; bopindolol and other diuretics; penbutolol and other diuretics; metoprolol and other diuretics; atenolol and other diuretics; atenolol and other diuretics, combinations; labetalol and other diuretics; timolol, thiazides and other diuretics; atenolol, thiazides and other diuretics; metoprolol and felodipine; atenolol and nifedipine; bisoprolol and amlodipine; nebivolol and amlodipine; metoprolol and amlodipine; propranolol and other combinations; sotalol and acetylsalicylic acid; metoprolol and acetylsalicylic acid; bisoprolol and acetylsalicylic acid; metoprolol and ivabradine; carvedilol and ivabradine; perindopril, bisoprolol and amlodipine; perindopril and bisoprolol |
| Calcium-antagonists | Amlodipine; felodipine; isradipine; nicardipine; nifedipine; nimodipine; nisoldipine; nitrendipine; lacidipine; nilvadipine; manidipine; barnidipine; lercanidipine; cilnidipine; benidipine; clevidipine; mibefradil; verapamil; gallopamil; diltiazem; fendiline; bepridil; lidoflazine; perhexiline; nifedipine and diuretics; amlodipine and diuretics; amlodipine and celecoxib; verapamil, combinations; nifedipine, combinations; enalapril and lercanidipine; lisinopril and amlodipine; perindopril and amlodipine; ramipril and felodipine; enalapril and nitrendipine; ramipril and amlodipine; trandolapril and verapamil; delapril and manidipine; perindopril, amlodipine and indapamide; perindopril, bisoprolol and amlodipine; ramipril, amlodipine and hydrochlorothiazide; metoprolol and felodipine; atenolol and nifedipine; bisoprolol and amlodipine; nebivolol and amlodipine; metoprolol and amlodipine |
| Diuretics | Bendroflumethiazide; hydroflumethiazide; hydrochlorothiazide; chlorothiazide; polythiazide; trichlormethiazide; cyclopenthiazide; methyclothiazide; cyclothiazide; mebutizide; bendroflumethiazide and potassium; hydroflumethiazide and potassium; hydrochlorothiazide and potassium; chlorothiazide and potassium; polythiazide and potassium; trichlormethiazide and potassium; cyclopenthiazide and potassium; methyclothiazide and potassium; cyclothiazide and potassium; quinethazone; clopamide; chlortalidone; mefruside; clofenamide; metolazone; meticrane; xipamide; indapamide; clorexolone; fenquizone; quinethazone and potassium; clopamide and potassium; chlortalidone and potassium; mefruside and potassium; clofenamide and potassium; mersalyl; theobromine; cicletanine; furosemide; bumetanide; piretanide; torasemide; furosemide and potassium; bumetanide and potassium; etacrynic acid; tienilic acid; muzolimine; etozolin; amiloride; triamterene; hydrochlorothiazide and potassium-sparing agents; trichlormethiazide and potassium-sparing agents; epitizide and potassium-sparing agents; altizide and potassium-sparing agents; mebutizide and potassium-sparing agents; chlortalidone and potassium-sparing agents; cyclopenthiazide and potassium-sparing agents; metolazone and potassium-sparing agents; bendroflumethiazide and potassium-sparing agents; butizide and potassium-sparing agents; furosemide and potassium-sparing agents; bumetanide and potassium-sparing agents; clorexolone, combinations with psycholeptics; chlorothiazide, combinations; hydroflumethiazide, combinations; hydrochlorothiazide, combinations; captopril and diuretics; enalapril and diuretics; lisinopril and diuretics; perindopril and diuretics; ramipril and diuretics; quinapril and diuretics; benazepril and diuretics; cilazapril and diuretics; fosinopril and diuretics; delapril and diuretics; moexipril and diuretics; zofenopril and diuretics; ramipril, amlodipine and hydrochlorothiazide; losartan and diuretics; eprosartan and diuretics; valsartan and diuretics; irbesartan and diuretics; candesartan and diuretics; telmisartan and diuretics; olmesartan medoxomil and diuretics; azilsartan medoxomil and diuretics; fimasartan and diuretics; candesartan, amlodipine and hydrochlorothiazide; olmesartan medoxomil, amlodipine and hydrochlorothiazide; oxprenolol and other diuretics; pindolol and other diuretics; bopindolol and other diuretics; penbutolol and other diuretics; metoprolol and other diuretics; atenolol and other; diuretics; atenolol and other diuretics, combinations; labetalol and other diuretics; timolol, thiazides and other diuretics; atenolol, thiazides and other diuretics |
| Glucose-lowering drugs (insulin and other glucose‑lowering drugs) | Insulin (any type); phenformin; metformin; buformin; glibenclamide; chlorpropamide; tolbutamide; glibornuride; tolazamide; carbutamide; glipizide; gliquidone; gliclazide; metahexamide; glisoxepide; glimepiride; acetohexamide; glymidine; phenformin and sulfonylureas; metformin and sulfonylureas; metformin and rosiglitazone; glimepiride and rosiglitazone; metformin and pioglitazone; glimepiride and pioglitazone; metformin and sitagliptin; metformin and vildagliptin; pioglitazone and alogliptin; metformin and saxagliptin; metformin and linagliptin; pioglitazone and sitagliptin; metformin and alogliptin; metformin and repaglinide; metformin and dapagliflozin; metformin and canagliflozin; metformin and acarbose; metformin and gemigliptin; linagliptin and empagliflozin; metformin and empagliflozin; saxagliptin and dapagliflozin; metformin and evogliptin; metformin and ertugliflozin; sitagliptin and ertugliflozin; metformin, saxagliptin and dapagliflozin; acarbose; miglitol; voglibose; troglitazone; rosiglitazone; pioglitazone; sitagliptin; vildagliptin; saxagliptin; alogliptin; linagliptin; gemigliptin; evogliptin; sitagliptin and simvastatin; gemigliptin and rosuvastatin; exenatide; liraglutide; lixisenatide; albiglutide; dulaglutide; semaglutide; dapagliflozin; canagliflozin; empagliflozin; ertugliflozin; ipragliflozin; guar gum; repaglinide; nateglinide; pramlintide; benfluorex; mitiglinide; tolrestat; sotagliflozin |
| Lipid-lowering drugs | Simvastatin; lovastatin; pravastatin; fluvastatin; atorvastatin; cerivastatin; rosuvastatin; pitavastatin; clofibrate; bezafibrate; aluminium clofibrate; gemfibrozil; fenofibrate micronized; simfibrate; ronifibrate; ciprofibrate; etofibrate; clofibride; choline fenofibrate; colestyramine; colestipol; colextran; colesevelam; niceritrol; nicotinic acid; nicofuranose; aluminium nicotinate; nicotinyl alcohol (pyridylcarbinol); acipimox; nicotinic acid, combinations; dextrothyroxine; probucol; tiadenol; meglutol; omega-3-triglycerides incl. other esters and acids; magnesium pyridoxal 5-phosphate glutamate; policosanol; ezetimibe; alipogene tiparvovec; mipomersen; lomitapide; evolocumab; alirocumab; lovastatin and nicotinic acid; simvastatin and ezetimibe; pravastatin and fenofibrate; simvastatin and fenofibrate; atorvastatin and ezetimibe; rosuvastatin and ezetimibe; simvastatin and acetylsalicylic acid; pravastatin and acetylsalicylic acid; atorvastatin and amlodipine; simvastatin, acetylsalicylic acid and ramipril; rosuvastatin and acetylsalicylic acid; atorvastatin, acetylsalicylic acid and ramipril; rosuvastatin, amlodipine and lisinopril; atorvastatin and acetylsalicylic acid; rosuvastatin and amlodipine; rosuvastatin and valsartan; atorvastatin, amlodipine and perindopril; atorvastatin, acetylsalicylic acid and perindopril; rosuvastatin, perindopril and indapamide; rosuvastatin, amlodipine and perindopril; atorvastatin and perindopril; bempedoic acid; rosuvastatin and omega-3 fatty acids; atorvastatin and omega-3 fatty acids; rosuvastatin and fenofibrate; rosuvastatin and fimasartan; rosuvastatin and ramipril |
| Proton pump inhibitors | Omeprazole; pantoprazole; lansoprazole; rabeprazole; esomeprazole; dexlansoprazole; dexrabeprazole; lansoprazole, combinations; rabeprazole, combinations; naproxen and esomeprazole |
| NSAIDs | Phenylbutazone; mofebutazone; oxyphenbutazone; clofezone; kebuzone; indometacin; sulindac; tolmetin; zomepirac; diclofenac; alclofenac; bumadizone; etodolac; lonazolac; fentiazac; acemetacin; difenpiramide; oxametacin; proglumetacin; ketorolac; aceclofenac; bufexamac; indometacin, combinations; diclofenac, combinations; piroxicam; tenoxicam; droxicam; lornoxicam; meloxicam; meloxicam, combinations; ibuprofen; naproxen; ketoprofen; fenoprofen; fenbufen; benoxaprofen; suprofen; pirprofen; flurbiprofen; indoprofen ; tiaprofenic acid; oxaprozin; ibuproxam; dexibuprofen; flunoxaprofen; alminoprofen; dexketoprofen; naproxcinod; ibuprofen, combinations; naproxen and esomeprazole; ketoprofen, combinations; naproxen and misoprostol; mefenamic acid; tolfenamic acid; flufenamic acid; meclofenamic acid; celecoxib; rofecoxib; valdecoxib; parecoxib; etoricoxib; lumiracoxib; polmacoxib; nabumetone; niflumic acid; azapropazone; benzydamine; proquazone; nimesulide; feprazone; morniflumate; feprazone, combinations |
| Antidepressants | Desipramine; imipramine; imipramine oxide; clomipramine; opipramol; trimipramine; lofepramine; dibenzepin; amitriptyline; nortriptyline; protriptyline; doxepin; iprindole; melitracen; butriptyline; dosulepin; amoxapine; dimetacrine; amineptine; maprotiline; quinupramine; zimeldine; fluoxetine; citalopram; paroxetine; sertraline; alaproclate; fluvoxamine; etoperidone; escitalopram; isocarboxazid; nialamide; phenelzine; tranylcypromine; iproniazide; iproclozide; moclobemide; toloxatone; oxitriptan; tryptophan; mianserin; nomifensine; trazodone; nefazodone; minaprine; bifemelane; viloxazine; oxaflozane; mirtazapine; bupropion; medifoxamine; tianeptine; pivagabine; venlafaxine; milnacipran; reboxetine; gepirone; duloxetine; agomelatine; desvenlafaxine; vilazodone; hyperici herba; vortioxetine; amitriptyline and psycholeptics; melitracen and psycholeptics; fluoxetine and psycholeptics; esketamine |
| *Elective PCI* |  |
| Elective PCI | PCI occurring without a diagnosis code of MI or unstable angina (ICD-10: I20.0; ICD-9: 411.1x) in the 30 days prior to (and including) the date of the PCI procedure |
| **Codes for inclusion and exclusion criteria** (not already defined above) | |
| Myocardial infarction hospitalization | ICD-10: I21-I23; ICD 9: 410, 429.7 |
| Anticoagulants | Vitamin K antagonists: dicoumarol, phenindione, warfarin, phenprocoumon, acenocoumarol, ethyl biscoumacetate, clorindione, diphenadione, tioclomarol, fluindione; Heparin group: heparin, antithrombin III, dalteparin, enoxaparin, nadroparin, parnaparin, reviparin, danaparoid, tinzaparin, sulodexide, bemiparin; heparin, combinations; Direct factor Xa inhibitors: dabigatran etexilate, rivaroxaban, apixaban, edoxaban;  Old (prior to 2013) rivaroxaban, betrixaban;  Direct thrombin inhibitors: desirudin, lepirudin, argatroban, melagatran, ximelagatran, bivalirudin |
| Severe hepatic impairment | ICD-10: I85.0, I85.9, I86.4, I98.2, K70.4, K71.1, K72.1, K72.9, K76.5-K76.7; ICD-9: 456, 572 |
| Renal failure requiring dialysis | ICD-10: Z49.1-Z49.2, Z99.2; ICD-9: V56.0, V56.8, V45.11; or country-specific dialysis procedure codes^d^ |
| Chronic Kidney Disease Stage 5 | ICD-10: N18.5; ICD-9: 585.5, 585.6 |
| *Strong CYP3A4 inhibitor* |  |
| Strong inhibitors | Ketoconazole; itraconazole; voriconazole; telithromycin; clarithromycin; nefazodone; ritonavir; saquinavir; nelfinavir; indinavir; atazanavir; esomeprazole, amoxicillin and clarithromycin; lansoprazole, amoxicillin and clarithromycin; lansoprazole, clarithromycin and tinidazole; omeprazole, amoxicillin and clarithromycin; pantoprazole, amoxicillin and clarithromycin; pantoprazole, amoxicillin, clarithromycin and metronidazole; rabeprazole, amoxicillin and clarithromycin; vonoprazan, amoxicillin and clarithromycin; darunavir and ritonavir; ombitasvir, paritaprevir and ritonavir; atazanavir and cobicistat; dasabuvir, ombitasvir, paritaprevir and ritonavir; lopinavir and ritonavir; atazanavir and ritonavir; ceritinib; cobicistat; darunavir and cobicistat; darunavir; emtricitabine, tenofovir alafenamide, darunavir and cobicistat; emtricitabine, tenofovir alafenamide, elvitegravir and cobicistat; emtricitabine, tenofovir disoproxil, elvitegravir and cobicistat; idelalisib; dasabuvir; posaconazole; mifepristone |
| Substrates with narrow therapeutic index | Cyclosporine; quinidine; simvastatin at doses >40 mg daily; lovastatin at doses >40 mg daily |
| Strong inducers | Rifampicin; phenytoin; carbamazepine; phenobarbital; rifampicin and isoniazid; rifampicin, ethambutol and isoniazid; rifampicin, pyrazinamide and isoniazid; rifampicin, pyrazinamide, ethambutol and isoniazid; phenytoin, combinations; fosphenytoin; primidone; enzalutamide; ivacaftor and lumacaftor; mitotane; apalutamide; pentobarbital; ivacaftor; ivacaftor and tezacaftor |

ACE, angiotensin-converting enzyme; ARB, angiotensin II receptor blocker; ARNI, angiotensin receptor neprilysin inhibitor; ASA, acetyl salicylic acid;
ATC-code, Anatomical Therapeutic Chemical code; CABG, coronary artery bypass graft; CHD, coronary heart disease; CKD, chronic kidney disease; COPD, chronic obstructive pulmonary disorder; CPRD, Clinical Practice Research Datalink; CPT, current procedural terminology; CV, cardiovascular; CYP3A4, cytochrome P450 3A4; GP, general practitioner; ICD, International Classification of Diseases; ICD-9, International Classification of Diseases 9^th^ Revision; ICD-10, International Classification of Diseases 10^th^ Revision; MACE, major adverse cardiovascular events; MI, myocardial infarction; MRA, mineralocorticoid receptor antagonist; NSAIDs, non-steroidal anti‑inflammatory drugs; ONS, Office for National Statistics; OPCS, Operating Procedure Codes; PCI, percutaneous coronary intervention.

^a^ Dilation of coronary artery; percutaneous transluminal coronary angioplasty with or without insertion of stent, or other recanalization of coronary artery; extirpation of matter from coronary artery; procedure or insertion of a certain amount of vascular stents, other removal of coronary artery obstruction; transcatheter placement of an intracoronary stent.

^b^ Connection to coronary artery from internal mammary artery; anastomosis or sequential anastomoses or other connection to coronary arteries from internal mammary arteries; anastomosis to coronary arteries from bilateral internal mammary arteries; connection or anastomosis or sequential anastomoses or other connection to coronary artery from gastroepiploic artery; aorto-coronary venous bypass (with or without single, two, three, four, five, or six distal anastomosis); other aorto-coronary venous bypass; aorto-coronary bypass using single or two prosthetic grafts; other aorto-coronary bypass using prosthetic graft; or coronary bypass using free arterial graft (of internal mammary artery or of gastroepiploic artery) or using free graft of radial artery or using other free arterial graft; abdominal-coronary artery bypass; open chest transmyocardial revascularization; heart revascularization by arterial implant; minimally invasive direct coronary artery bypass surgery involving mini-thoracotomy or mini-sternotomy surgery.

^c^ Exarticulation of hip, amputation of femur, other amputation or related operation on hip or femur, exarticulation of knee, amputation of lower leg, other amputation or related operation on knee or lower leg, exarticulation of ankle or foot, talocrural exarticulation, intertarsal exarticulation, tarsometatarsal exarticulation, transmetatarsal exarticulation, metatarsophalangeal exarticulation, partial toe exarticulation, amputation of ankle or foot, talocrural amputation, intertarsal amputation, tarsometatarsal amputation, transmetatarsal amputation, metatarsophalangeal amputation, partial toe amputation, talocrural amputation, intertarsal amputation, tarsometatarsal amputation, transmetatarsal amputation, metatarsophalangeal amputation, partial toe amputation, and other amputation or related operation on ankle or foot; detachment of femoral or knee region, upper leg, lower leg, feet or toes; traumatic amputation of toe, foot or leg; revision of amputation stump; lower limb amputation status; disarticulation of hip, knee, or ankle; hemipelvectomy.

^d^ Hemofiltration; hemodialysis; hemodiafiltration; hemoperfusion; dialysis procedure due to lack of function and failure of a kidney transplant; peritoneal dialysis; renal dialysis status; extracorporeal dialysis; other dialysis; performance of urinary filtration; irrigation of peritoneal cavity; fluoroscopy of dialysis shunt/fistula; venous catheterization for renal dialysis; arteriovenostomy for renal dialysis; percutaneous transluminal mechanical thrombectomy and/or infusion for thrombolysis, and dialysis circuit; transluminal balloon angioplasty.

**Supplementary Table 2**. **Details of the study organization**

| **Study organization** |
| --- |
| Study sponsor: AstraZeneca  External scientific committee:   - Responsible for:   - The planning, development and scientific integrity of all publications and presentations derived from the ALETHEIA study in collaboration with AstraZeneca   - The content and implementation of the study protocol and analysis plan, its interpretation, and reporting of the study results - Members:   - Marc Bonaca (chair), MPH, CPC Clinical Research/CPC Community Health, Aurora, Colorado, United States   - Albert Ariza-Solé, PhD, Hospital Universitari de Bellvitge, Barcelona, Spain   - Evangelos Giannitsis, PhD, University of Heidelberg, Heidelberg, Germany   - Tomas Jernberg, PhD, Danderyd Hospital, Karolinska Institute, Stockholm, Sweden   - Aldo P Maggioni, PhD, ANMCO Research Center, Florence, Italy   - Robert F Storey, PhD, University of Sheffield, Sheffield, United Kingdom   - Jurrien ten Berg, PhD, St Antonius Hospital, Nieuwegein, Netherlands   Coordinating center: Evidera   - Responsible for the study oversight and coordination of analyses across all databases, as well as the conduct of analyses for US, UK, and Swedish data.   Research partners: Ingress Health (Germany), ReS (Italy)   - Responsible for conduct of analyses in respective countries. |
| **Processes for data analyses**   - As the datasets used in this study are not the property of AstraZeneca or Evidera, the database holders will be responsible for data extraction. For datasets where the data can be analyzed directly by Evidera, individual-level data will be transferred to Evidera researchers. - Evidera researchers will analyze individual-level data when possible or will provide scientific and operational oversight with the data holders who will perform the analyses when individual-level data cannot be transferred to Evidera. - Evidera researchers will perform the pooling and meta-analysis of database level summary results. |
| **Processes for harmonization**   - Evidera researchers will be responsible for preparing code lists and table shells for each database. When data analyses will performed by a research partner, they will support in the adaptation of database specific code lists under the oversight of Evidera researchers. - Evidera researchers will be responsible for cross-country QC of code lists, table shells, database level results and pooled and meta-analyzed study results to ensure consistency and harmonization across all databases. |

QC, quality control; ReS, Fondazione Ricerca e Salute; UK, United Kingdom; US, United States.
